# Polycrystalline Films of Indium-Doped PbTe on Amorphous Substrates: Investigation of the Material Based on Study of Its Structural, Transport, and Optical Properties

**DOI:** 10.3390/ma17246058

**Published:** 2024-12-11

**Authors:** Jürgen Jopp, Vadim Kovalyuk, Elias Towe, Roni Shneck, Zinovi Dashevsky, Mark Auslender

**Affiliations:** 1 Ilse Katz Institute for Nanoscale Science & Technology, Ben Gurion University of the Negev, Beer Sheba 8410501, Israel; jjurgen@bgu.ac.il; 2 Department of Physics, Moscow State Pedagogical University, 119992 Moscow, Russia; 3 Departments of Electrical and Computer Engineering, and Materials Science and Engineering, Carnegie Mellon University, Hamerschlag Hall, 5000 Forbes Ave, Pittsburgh, PA 15213, USA; 4 Department of Materials Engineering, Ben Gurion University of the Negev, Beer Sheba 8410501, Israel; 5 School of Electrical and Computer Engineering, Ben Gurion University of the Negev, Beer Sheba 8410501, Israel

**Keywords:** lead telluride (PbTe), polycrystalline films, columnar grains, polycrystal growth methods (PVD), structural characterization (XRD, SEM, TEM, EDXS, HAADF), transport properties measurements (Hall effect, conductivity, thermopower), optical characterization (IR spectroscopy)

## Abstract

Nowadays, polycrystalline lead telluride is one of the premier substances for thermoelectric devices while remaining a hopeful competitor to current semiconductor materials used in mid-infrared photonic applications. Notwithstanding that, the development of reliable and reproducible routes for the synthesis of PbTe thin films has not yet been accomplished. As an effort toward this aim, the present article reports progress in the growth of polycrystalline indium-doped PbTe films and their study. The introduction foregoing the main text presents an overview of studies in these and closely related research fields for seven decades. The main text reports on the electron-beam-assisted physical vapor deposition of n-type indium-doped PbTe films on two different amorphous substrates. This doping of PbTe is unique since it sets electron density uniform over grains due to pinning the Fermi level. In-house optimized parameters of the deposition process are presented. The films are structurally characterized by a set of techniques. The transport properties of the films are measured with the original setups described in detail. The infrared transmission spectra are measured and simulated with the original optical-multilayer modeling tool described in the appendix. Conclusions of films’ quality in terms of these properties altogether are drawn.

## 1. Introduction

Lead salts, or lead chalcogenides (LCs) PbX, where X = S, Te, and Se, are semiconductors, crystallizing in a cubic rock-salt structure, that have direct narrow band gaps of 0.41eV, 0.32eV, and 0.29eV, respectively, at temperature T=300K. Furthermore, such ternary LCs as PbSe*_x_*S_1−*x*_ and PbSe*_x_*Te_1−*x*_, as well as lead–tin chalcogenides (LTCs) Pb_1−*y*_Sn*_y_*X with X = Te, exhibit full solid solubility while keeping the NaCl structure like binary LCs, and the same is valid for LTC with X = Se at 0<y≤0.4. No LTC exists at X = S, but we extend the notation, implying y=0 for PbS. These materials have been studied in depth, and many of their properties and device applications have been well documented over a long time; see, e.g., [1,2,3,4,5] and references therein. The main applications of the LCs and above more-involved compounds on their base have been and remain in the fields of thermoelectric (TE) and infrared (IR) photonic devices.

The materials in question are among the premiere-performing ones in terms of the TE figure of merit (ZT); see the definition, e.g., in ref. ([6] Chapter 1), and, in the mid-*T* range of 500–900 K, see ibid. as for 2006, and in reviews over the last decade devoted to broad classes of thermoelectrics [7,8,9,10] and those focusing on the PbTe-based compounds [11,12,13,14,15]. For this reason, since the 1960s, LTCs have been one of the leading applied materials (second only to ${\mathrm{Bi}}_{2}{\mathrm{Te}}_{3}$-based ones) in designing commercial TE generators (TEGs) for terrestrial applications ([6] Chapter 9) and space missions ([6] Chapter 43) [9,12,16]. A long history of powering several NASA spacecraft by PbTe-based TEGs may be learned, e.g., in the reviews [9,12]. The development of TE physics and devices was pioneered in the USSR in the 1930s, and the first practical TEGs were distributed, starting from 1941, as part of the power supply to a “partisan mess kit“ during WW II ([6] Appendix I; Chapter 1).

It is not known whether LTCs were employed in early Soviet TEGs, yet exploring the photoconductivity of PbS began in Germany in the early 1930s, aiming at the design of IR photon detectors (IR-PDs) [17]. Then, in about 1943, the photoconductive (PC) PbS cells were brought to the manufacturing stage; the first practical IR-PDs on their base were deployed for a variety of military applications used by the German army in the rest of WW II, such as an airborne IR system with superior detection range ([18] Section 2). Overviews of the continuation of those research studies and development in the allied powers after 1945, including the discovery of photoconductivity in PbTe and PbSe and their adaptation similar to that occurring with PbS to the IR PC detectors (PCDs) design in the US, can be found in, e.g., ([1] § 1), ([2] Section 2) and, over a wider historical perspective, in ref. ([18] Section 2).

As with any materials, the structural form of LTCs has a crucial impact on the performance of devices manufactured on their base. In this introduction, we regard bulk crystals and single-crystalline and polycrystalline films while omitting low-dimensional structures that lie out of our scope. Choosing a specific structural form is subject to the application, spectral and *T* ranges, and other device operation and size demands. Bulk-crystalline LTCs are obtained with a method suitable specifically to each of them and chosen from established melt and vapor crystal growth techniques. Single-crystal films became available with the advent of modern film growth techniques, such as liquid-phase epitaxy (LPE), hot-wall epitaxy (HWE), and molecular-beam epitaxy (MBE) and their specific adaptations. Regarding the structure, the earliest used LCs were probably the polycrystalline films of PbS obtained with various techniques. These include chemical ones, such as chemical bath deposition (CBD), also known as chemical solution deposition [19], chemical vapor deposition (CVD) [20], and physical vapor deposition (PVD) [21], which, today, some authors call, not quite accurately, vapor phase deposition.

During or after the preparation, LTCs can be doped with suitable impurity atoms such as Cl, Br, and In or Li, Na, K, and Tl in order to have n- or p-type conductivity, respectively, in the doped specimens. Preparing crystals and epitaxial layers of LTCs that are quite large and thick, respectively, with a high degree of perfection, stoichiometry, and homogeneity of the alloy components and impurity distributions is challenging [3,4,5], ([6] Chapter 20). Different kinds of lattice defects, such as interstitial and anti-site ones, vacancies, and dislocations, may persist after the fabrication. In polycrystalline films’ syntheses, similar problems arise regarding crystallites, i.e., crystalline grains. For these films, the above issues are joined by other specific ones, such as non-homogeneity of grain size distribution and film thickness, texture, and inter-grain voids. All the challenges are gradually met by improving the technologies, but it proves especially difficult for polycrystalline film fabrication. Unless the defects and granular structure block the heat and charge transfers in the targeted substances, they may not preclude TE devices based on these films from working. Thus, to these ends, LTCs are usable in all three above-noted structural forms in principle and prove so indeed, when provided topmost increasing ZT of the selected material with intelligent engineering of their microstructure [22], defects [23] and dislocations [24,25], phonon and electron transport [24,26,27], and electronic band structure [14,15,28,29,30] by managing the simulations, synthesis, and doping, respectively ([6] Chapter 48), [15,31]. Thanks to TE physics and the above material science prerequisites, the field of LCs/LTCs-based TE devices has become well matured these days [32].

The situation with the development and advantages of IR photonic devices based on LTCs is not so clear-cut. Until the early 1990s, LTCs and mercury–cadmium telluride (MCT) Hg_1−*x*_Cd*_x_*Te [33,34], which both have a narrow band gap tuned with the alloy composition, were in serious competition for prevalence among semiconductors photo-responsive in IR. At first, PCDs of polycrystalline LCs films, developed in the 1940s to 1950s [1,2,18], competed with similar MCT ones that emerged later, and, at that time, the former IR-PDs dominated the market. The peak sensitivity benchmarks attainable with polycrystalline LCs-based PCDs are similar to those of the matching MCT and III-V compounds. At the same time, the latter function at 77K, whereas PCDs of polycrystalline PbS and PbSe work uncooled, and those of polycrystalline PbTe require only a minor cooling below room temperature (RT). Such a doubtless advantage is, to some extent, traded off by the intricacy and multi-variance of the technologies for preparing the PC films of polycrystalline LCs.

While remaining undisclosed in detail, the recipes of the 1940s for the PbS PC film deposition and related PCD fabrication described empirical though well-tried CBD. Still, starting in 1946, based on incomplete information, researchers in many places began to rediscover CBD of PbS films, while continuously improving, advancing, and extending these to other LCs ([1] § 6, 7),( [19] Chapter 5), and also applying CVD for that purpose ([20] Chapter 12). In parallel, developing PVD and growth from melt specifically for PbS and other LCs emerged ([1] § 2), ([2] Section 8), and ([3] Chapter 1, §1.2), respectively, and has continued until now. Though, generally, CBD outperformed others in terms of better film uniformity and more stable and reproducible results in the initial stage, the evaporation techniques have attained equal footing with CBD. Over time, PVD has become the preferred film-deposition method for avoiding excessive chemistry in a lab. Its advantages over epitaxy are a low cost and the ability to use more diverse substrates. Ones such as mica and BaF_2_ [35], which acquired a wide use, e.g., in MBE of LCs [36], quartz glass, and thermally oxidized Si [37]—see also a review [38]—to mention a few, were successfully utilized.

Unlike the foolproof usage in TE devices, the polycrystalline films of LCs, as grown with the above-noted prerequisites, are not directly usable in PCDs because of a lack of or very low photosensitivity. It was speculated in the past, and confirmed by later studies, that such insensitivity is due to a small grain size, e.g., for CBD, typically of 0.1–1.0 μm. For this reason, the key issue for preparing high-performance PCDs is photo-sensitizing the films. This is achieved with oxidation, which, e.g., in CBD, may be carried out by adding suitable chemicals in the deposition bath during the process, post-process heat treatment in the oxygen atmosphere, or chemical oxidation of the as-grown film. Then, little information was reported on the details of the photo-sensitization processes, and they varied much with staff who made a few samples in labs and batches of ready IR-PDs in companies. Later, those details were in-depth reported for CBD [19], and suitable in- and post-deposition oxidation processes were also developed for the CVD [20] and PVD [38]. At the dawn of using the polycrystalline films of LCs for PCDs, several theories explaining the sensitizing effect of the oxidant, some of which contradicted each other, were put forward; see an overview in ref. [1,2] and, for a more recent and consistent theory, in ref. [38].

With the emergence of epitaxy methods, great hopes arose for avoiding complicated fabrication routes for the above polycrystalline films and, while understanding their sensitization and carrier recombination mechanisms is until now incomplete, manufacturing IR-PDs and quantum-effect devices using their high-quality epitaxial counterparts [2,39,40,41]. Then, a belief came that the epitaxial LCs-based devices would be highly efficient in particular; thus, fabricated IR-PDs would retain the operation efficiency, similar to that of cooled MCT-based PCDs at RT, or at least well above the liquid nitrogen temperatures. The reasons for that belief are the advantages of LTCs over the MCT under similar conditions, such as their band gap and operating temperature. An essential one is the orders-of-magnitude smaller Auger coefficient, weakly varying with the temperature at 77K≤T≤295K as was predicted theoretically [42,43,44] and confirmed experimentally [45,46,47]. Such superiority of LTCs also holds over those III–V semiconductors, which obey the above-noted restraints [48], ([49] Chapters 7, 8; [50] Chapters 3, 4, 6). However, the epitaxial growth of LTCs drastically slowed down because of their notable drawbacks, such as thermal and lattice mismatches with the most crystalline substrates, except for CaF_2_, BaF_2_, NaCl, KCl, and mica (for MBE, see [36]). Yet, except for CaF_2_, these are impractical as they react with water and hardly attach to contacts. Still, at the end of the 1980s, a group at ETH, Zurich, successfully propelled the MBE growth of such epitaxial films on crystalline silicon (Si) using CaF_2_/BaF_2_ buffers to overcome the mismatch problems [51]. Since then, these problems have become no longer fundamental, and the ETH team continued to pursue this technique for preparing optoelectronic devices—see [36,51]—in particular, IR-PDs, based on those films. Such significant progress was made that, if these developments took a shorter time, the LCs IR-PDs would seriously compete with MCT-based ones regarding manufacturing ease, homogeneity, and cost. But, MCT technology developed faster and, eventually, it won the race [17,34], while transferring knowledge on LTC epitaxy to semiconductor fabs stopped in the 1990s.

At the same time, attempts to prepare PC polycrystalline films of LTCs and uncooled IR PCDs on their base never ceased, despite the above concurrent complications, and, for the last two decades, interest in this issue has been reviving and steadily growing. Within this connection, the research for working out reliable, well-tried, and reproducible paths for preparing those films has continued and is being trained—see, e.g., in refs. [19,20,38]—as of 2006. Since then, ongoing reports on the deposition of polycrystalline LTCs have appeared. A list of materials, not pretending to be complete by far, includes Pb_1−*y*_Sn*_y_*Te (y=0,0.1,0.2) [52,53,54,55,56,57,58], PbSe_1−*x*_S*_x_* (x=0.23,0.42), PbSe_1−*x*_Te*_x_* (x=0.12,0.49) [59], and PbSe [60,61,62,63,64]. The known deposition techniques, such as CBD [62,63,64,65] and PVD [52,53,54,55,56,57,58,59,66,67], which were resorted to, have been revisited and, in some cases, modified using increasingly advanced miscellaneous instrumental equipment for film fabrications and characterizations. For PCD and photo-luminescence applications, a fabrication should eventually include in- or post-synthesis photo-sensitization treatment by oxidation noted before, iodization [38,53,54,58,59,62,63,64,66,67], and concurrent laser annealing [64]. In this framework, highly efficient uncooled IR PCDs on the base of polycrystalline LCs have been demonstrated, such as resonant cavity-enhanced (RCE) ones using PbTe [68,69], and PbSe [67], as well as monolithic ones using PbSe [56,65,66,70] and ternary LCs [59]. PbSe PCDs have been assessed for use in IR optical communication [71], imaging [63], and sensing [67], and underlying thin films have been examined in depth about the diffusion of minority carriers [35,72], energy level diagrams [62], and mechanisms of photo-luminescence [58,60] and photoconductivity [73].

By the ZT definition, the direct strategy used to increase it includes concurrently reducing thermal conductivity ϰ and enhancing both Seebeck coefficient *S* and conductivity σ and, in this aspect, tellurides among LTCs occupy a special place. The decrease in ϰ is achieved by creating high-density defects such as grain boundaries, static strains, and dislocations (due to nano-crystalline patterning) that strongly scatter heat-carrying phonons. The increase in *S* and σ is attained by preparing ternary and quaternary alloys of PbTe with small amounts of Sn, Se, and S (with no Sn), its composites with suitable binary compounds for smart engineering of the band structure, and heavy doping for providing a high concentration of current carriers. It is these types of processing altogether that proved capable of increasing ZT well above unity for diverse PbTe-based compounds, showing notable progress over the last few decades [6,7,8,9,10,12,13,15,23,26,31]. By now, record maxima of ZT attained for the p- and n-type PbTe-based materials are ${\left(ZT\right)}_{\max}=2.57$ at $T_{\max}=850\;K$ and ${\left(ZT\right)}_{\max}=2.20$ at $T_{\max}=800\;K$, respectively; see the table in review [15], summarizing most recent data in this field. Also, doped chalcogenides of IV group elements other than Pb, such as, e.g., SnSe and GeTe, are expediently worth noting in the low- and medium-*T* TE applications perspective. Recent deep studies documented the synthesis and investigation of such thin-film TE materials from this class as a polycrystalline Sn_1−*x*_Ag*_x_*Se [74] and Te-deficient epitaxial GeTe on a Si substrate [75]. For x=0.02, Ref. [74] reported ZT=0.93 at T=550K, which is a record value for such *T*. In ref. [75], authors realized a smart combination of phase-domain engineering, to uncouple the transport of phonons and carriers, and point defects control to suppress the formation of Ge vacancies, which create a high holes concentration *p* over $10^{21}\;{\mathrm{cm}}^{-3}$, thus impairing ZT. For $p=2.8\times 10^{19}\;{\mathrm{cm}}^{-3}$, they report ZT=0.26 at T=400K, which is a good value given a low *T* and still rather high *p*. About the same result was reported for the thin films of Ge_0.96_Bi_0.04_Te [76].

In contrast with the above high TE performance of PbTe-based materials, monolithic PCDs based on LCs, in which Te content dominates that of other chalcogens, lag in RT photosensitivity behind those on the base of LCs, containing only S and Se, or Se and Te, with a relatively small content of the latter [59], or only Se [56,62,64,65,66,70]. This drawback can be eliminated by moderate cooling down to a temperature twice as high as liquid nitrogen. When avoiding cryogenics, one can use the RCE-PD design, where a photosensitized PbTe film is properly integrated with a multilayer environment [68,69]. Among LCs, PbTe notably stands out from viewpoints of both pure physics and polycrystalline-film technology. It has such striking quantitative differences with PbS and PbSe, having a qualitatively similar band structure, a much stronger anisotropy and non-parabolic warping of constant-energy surfaces, and a more intimate closeness of main and subsidiary valence bands, with tops at the points *L* and Σ, respectively, of the Brillouin zone [3,4,5,77]. Technologically, the PbTe films can be grown in a close-packed columnar grain structure with grain sizes from tens to hundreds of nanometers [37,38,52,53,54,57,68,72], in marked contrast with the PbS and PbSe films, which finally form flake grain structures with many pores [38,56,60,61,62,63,64,66]. High porosity makes the effective refractive index (RI) of the above films low [61], which significantly reduces the perspectives of their use in resonant metamaterial-based PDs relying on high RI contrast.

In optoelectronic devices, a high carrier concentration homogeneity is imperative for low-noise operation at high temperatures. However, a technological obstacle to this end in the case of LCs follows from special features of their phase diagrams, which result in the difficulty in preparing uniform crystals (or crystallites in the films) of near stoichiometric composition. Long-term studies evidenced that the most suitable method for synthesizing the best-purity, maximally stoichiometric crystals of LCs is their growth from the vapor phases ([3] Chapter 1, §1.4). Maybe for this reason it is PVD that produces crystallites in polycrystalline LCs with structural and chemical properties similar to those of crystalline ones. But, even in the best case, the equilibrium density of electrically active intrinsic defects is $10^{18}$–$10^{19}{\mathrm{cm}}^{-3}$, which releases free carriers of comparable concentrations. As the static dielectric constants of LCs are notably high, especially for PbTe, those electrons or holes do not freeze out at lowering *T*, and their densities, *n* or *p*, respectively, are highly spatially non-uniform. Even after annealing and doping with the above-noted atoms of halogens or alkaline metals, the homogeneity of LCs does not radically improve [3,4,78]. A well-established unique solution to this problem is doping LCs with In or Tl, resulting in n- or p-type samples with highly uniform *n* or *p*, respectively [78,79,80].

It appears that the In- and Tl-doping of PbTe allows for a much better tuning of Fermi level position, and hence controlling the electric and thermoelectric transport, than such doping of PbS and PbSe [78,79,80]. For the latter, the film deposition technology, with regard to film integrity, seems to arrive currently at an exhaustion stage [18,56,61,63,66], while integrating extrinsic doping into it is nearly impossible. In contrast, preparing the PbTe films has promising perspectives, since the In- and Tl-doping of PbTe can be well merged with PVD. Thus, the above distinctive physics and technology allow for a smart band structure and doping engineering to improve the properties of the PbTe films, which are crucial for the high efficiency of their device application [28,29,30]. For the eventual success in allowing for highly efficient and competitive PbTe-PCDs, further advancement and regime optimization for syntheses of polycrystalline PbTe films doped with In and Tl are of keen importance.

Above, we extensively reviewed the research field of interest from historical and current-state views. In the main text, we focus only on polycrystalline films of In-doped PbTe, notated below as PbTe〈In〉. Our study here is restricted to the materials science perspective, not concerning any applications. In Section 2 and Section 3, we report progress in advancing the synthesis, characterization, and properties analysis of this material. Section 4 offers a detailed discussion of the issues, and Section 5 concludes the study.

## 2. Materials and Methods

### 2.1. Samples Fabrication

The synthesis of PbTe〈In〉 was carried out by direct melting of its high-purity components for 10 h at the temperature of 1073K in sealed quartz ampules pumped out to a residual pressure of $10^{-5}$ millibar. Afterward, the ampules were taken from the furnace and separately quenched in cold water. The thus-obtained ingots were crushed into fine powders by ball milling in an Ar atmosphere. The final step was the preparation with these powders of a batch of the PbTe〈In〉 polycrystalline films. To this end, we employed an updated in-house PVD version with two substrates, as described below. In addition to the PVD advantages over the chemical methods noted in the introduction, its relative simplicity is worth mentioning. It makes PVD, generally speaking, a most accurately predicted process from a feature-scale simulation viewpoint. In addition, PVD is compatible with the full range of standard lithographic techniques, e.g., liftoff, which enables patterning the PVD-grown films at sufficiently small length scales suitable for many applications such as, e.g., integrated IR photonic circuits.

The films under study were obtained by electron-beam assisted PVD (EBPVD) using an Edwards E306A thermal evaporator through a rigid metal mask at a vacuum ∼$10^{-6}\;\mathrm{Torr}$ of residual pressure. Two commercially available amorphous substrates were tried: (i) the amorphous silicon dioxide a-SiO_2_ (a-SOX), the layer that was the top of the raw structure for silicon-on-insulator (SOI) technology, i.e., a Si (100) wafer, thermally oxidized on both sides [81]; (ii) Kapton^®^ polyimide (KPI) film [82]. Nominal thicknesses of each a-SOX layer, unoxidized Si bulk, and KPI film were 1.0μm, 368μm, and 100μm, respectively. The growth rates, controlled by a piezoelectric sensor, were kept at levels of 0.3–0.4 nm/s and 0.1–0.2 nm/s for the a-SOX and KPI substrates, respectively. Thus, by setting deposition times, films of various thicknesses were grown. Several substrate temperatures ($T_{s}$), controlled during the evaporation to within ±1°, were tested to pick up samples with the best possible integrity and large mean size of crystalline grains (crystallites). The $T_{s}$ were in the range of −50 °C to 350 °C and 250 °C to 365 °C for the a-SOX and KPI substrates, respectively. The post-growth processing of the films was carried out in a specially constructed vacuum-sealed stainless-steel case, placed in an oven. After pumping out the oven down to $10^{-3}$ millibar of residual pressure and Ar washing, some as-grown films were put into the oven with the argon (Ar) atmosphere at an excess pressure of ∼1atm and temperature of ∼400 °C. The films’ annealing lasted for 3 h, and the whole cycle until complete cooling took 24 h.

### 2.2. Samples Characterization

The structure, morphology, and chemical–elemental composition of the films grown were studied. For these purposes, we used X-ray diffraction (XRD), scanning electron microscopy (SEM), transmission electron microscopy (TEM), and atomic force microscopy (AFM). Both employed electron microscopes are equipped with energy-dispersive X-ray spectroscopy (EDXS), and the TEM instrument with high-angle annular dark field (HAADF) imaging facilities.

For the XRD, such X-ray diffractometers as Brucker D8-Advance (Brucker AXS GmbH, Karlsruhe, Germany) and Rigaku Model-2000 (Rigaku Corp., Tokyo, Japan) were employed, equipped with a graded parabolic mirror, shaping the beam to have an angular divergence of less than 0.02°, and use radiation produced by Cu $K_{\alpha }$ line (λ=1.5405 Å). Both common XRD θ:2θ scans and rocking curves were obtained. Secondary (reflected and transmitted) electron images were taken by JEOL JSM-7400F (JEOL Ltd., Tokyo, Japan), FEI Quanta 200™ E-SEM (FEI Co., Hillsboro, OR, USA) for high-resolution SEM (HRSEM) with accelerating voltages in the range of 0.1–30 kV, and JEOL JEM-2100F (JEOL Ltd., Tokyo, Japan) for high-resolution TEM (HRTEM) and scanning TEM (STEM) with a constant voltage of 200kV. In the case of the KPI substrate, HRSEM was performed under the accelerating voltage limited to 3kV, as KPI might melt at higher voltages. In contrast, the SOI wafer would withstand notably higher voltages. For the TEM imaging, a pair of very thin laminae of 0.1μm thickness with orientations close to [001] and [111], characteristic of the crystalline PbTe growth, were cut from some films. An AFM apparatus, Autoprobe CP (Park Systems Inc., Santa Clara, CA, USA), was employed to study the morphology and topography of the films’ surfaces.

Also, the transport properties of the fabricated films, including the electrical conduction, Hall effect, and thermoelectric effect, were investigated. To this end, such key physical quantities as σ, *S*, and Hall coefficient $R_{H}$ were measured over the various temperature ranges depending on the given quantity and substrate type. In the a-SOX substrate cases, σ and $R_{H}$ were measured at 80K≤T≤300K and permanent magnetic fields with the induction *B* up to 1.0T, whereas *S* at T=300K and B=0T. For samples with the KPI substrate, the temperature varied from 10K to 300K, and *B* up to 0.8T. Ohmic contacts for the conduction and Hall geometries (Au, Ni, or Cr pads) were prepared by thermal evaporation via an additional metal mask; σ and $R_{H}$ were measured to within 6% and 8% relative accuracy, respectively. The stability range of the magnetic field measurements was within ±3%. For experimenting below RT, we employed an original setup that includes the closed-cycle Gifford-McMahon SRDK-101D cryocooler; for details, see [31,57]. To have enough room for non-constrained sample allocation in the cryocooler, we reduced the number of wires, and thus the thermal load. To this end, we elaborated on an electrical switch that allows the multimeter to be swapped between the Hall and the potential type of contact. The heat flow from the magnet manipulator limited the lowest achieved temperature (10K). The temperature was kept stable to within an accuracy of ±0.1K using the Cryo-con^®^ Model 32B controller (Cryo Con Systems, Inc., Rancho Santa Fe, CA, USA).

Finally, an acquired SOI wafer and a few fabricated PbTe〈In〉 films on a-SOX were characterized optically with the IR transmission spectroscopy. The transmission spectra were measured by a monochromator-based spectrograph ORIEL 77298 (Newport Corp., Irvine, CA, USA) with an appropriately tuned resolution. To provide a sufficient removal of fringes—see Appendix B.3—the spectrometer resolution was set as larger than an estimated fringes period. Non-monochromatic and stray radiation, inherent to any optical setup, were checked to have the least effect. The spectral transmittance data acquisition from the setup was performed using the LabVIEW software v2020 SP1 (National Instruments Corp., Austin, TX, USA).

### 2.3. Experimental Data Processing Using Images and Theoretical Models

The films’ volumetric structure and surface topography were concluded and visualized with the HRSEM and STEM images. The detailed surface morphology and RMS roughness were assessed from the AFM scans. The EDXS analysis provides information on the chemical composition of the synthesized films, including the In content *x*. Moreover, STEM HAADF imaging allows for portraying the spatial distribution of the alloy constituents and their possible segregation at interfaces and/or dislocations. The film texture was inferred directly from the XRD θ:2θ scans. The dislocations’ density *D* was estimated by processing the rocking curves with the method described in detail in Appendix A and the HRTEM images in the cases of a-SOX and KPI substrates, respectively.

The measured electric and thermoelectric characteristics of the films, viz. σ, $R_{H}$ and *S*, respectively, prove explainable, at least semi-quantitatively, in the one-electron theory of semiconductors framework, with a proper account for the band structure of PbTe and specificity of the impurity state created in it due to the In atoms alloying; see, e.g., recent paper [28] and references therein, and discussion in Section 4.2.

To account for the measured spectra of transmission through the SOI wafer (trilayer) and PbTe〈In〉 films on this substrate (tetra-layer), we simulated the spectra with the theoretical model developed in Appendix B.2. To this end, we used the model expressions of *T*, derived for the above two structures, and processed them to obtain $T_{a}$ due to Appendix B.3, since one layer in the structure is a slab. Obtaining the initial formulas with a widespread Airy method of coherent summation of transmission amplitudes is too tricky even for the trilayer and nearly impossible for the tetra-layer. A similar problem emerges in the presence of the slab, while summing the powers of incoherent rays, impinging the slab and being multiply reflected in it, has to be invoked. When performing the simulations, we plugged into them the nominal thicknesses $d_{i}$ of the constitutive layers and open-source data for the optical permittivity spectra of a-SOX and Si and non-doped PbTe crystals. No fitting with regard to $d_{i}$, and correcting the dispersion formulas, in particular, including the Drude term for n-PbTe, were carried out. Section 4.3 presents a discussion of the optical study.

## 3. Results

For all grown PbTe〈In〉 films, the EDXS analysis established that *x* is about the same as in the probed evaporation sources. For the a-SOX substrate, it was x=0.005 (0.5at.%), whereas, for the KPI one, *x* was the same and also x=0.01 (1.0at.%).

### 3.1. Structural Properties

**Figure 1:**
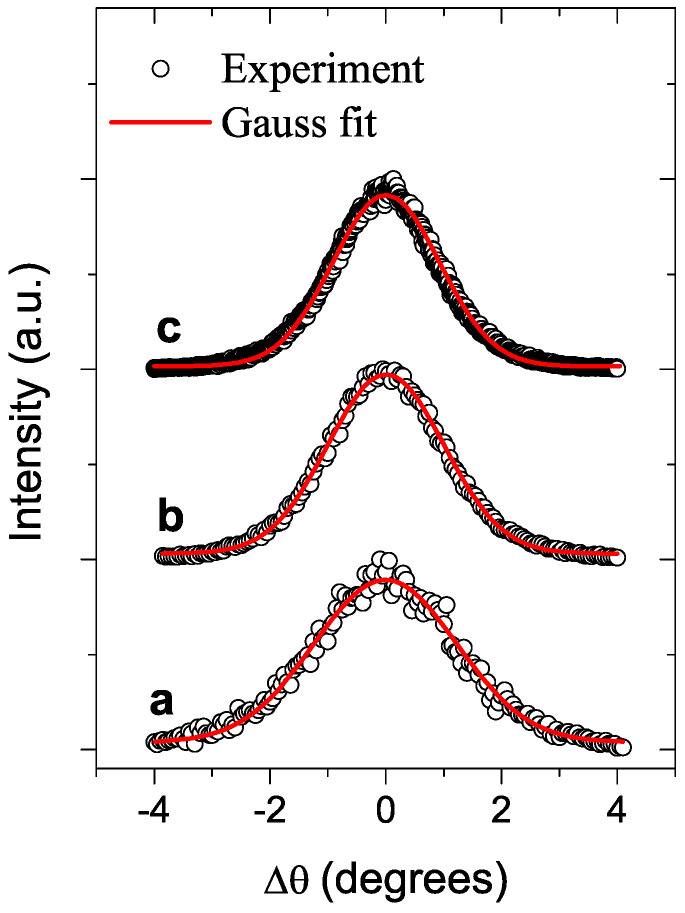
XRD rocking curves for three specimens presented in Table 1. Symbols (○) and red lines are the XRD data and fit lines, obtained with the fitting procedure as explained in Appendix A, respectively. The letter markers refer to the specimens—**a**: SOX−1; **b**: SOX−2; **c**: SOX−3.

**Figure 2:**
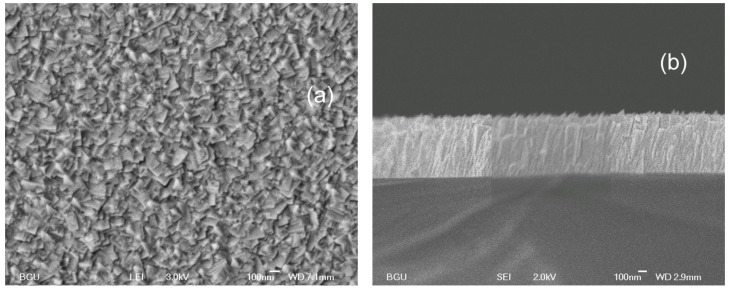
HRSEM images of the 1.0μm thick and 135nm average grain size PbTe〈In〉 film, grown with the nominal In content of 0.5at.% on the a-SOX substrate at $T_{s}=-50$ °C (not listed in Table 1): (**a**) top view; (**b**) cross-sectional view.

**Figure 3:**
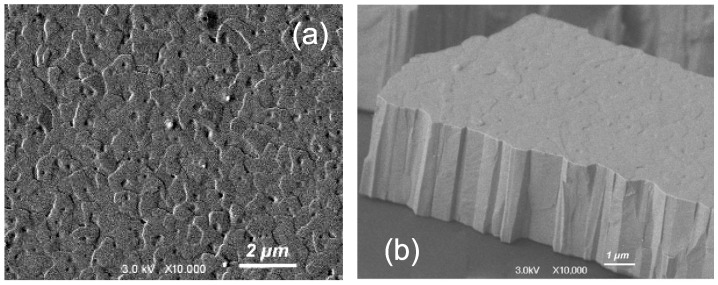
HRSEM images of the specimen KPI−5 itemized in Table 2: (**a**) top view; (**b**) cross-sectional/3D view.

Several structural parameters of PbTe〈In〉 films grown on the a-SOX (atop the SOI wafer) and KPI substrates are shown in Table 1 and Table 2, respectively. SEM imaging was used to finally adjust film thicknesses (*d*), assessed first by the growth rate and preassigned deposition time, and to determine grain sizes (*w*); see in Table 1 and Table 2. The cross-sectional- and top-view HRSEM images were specially processed to determine *d* and *w*, respectively. For sample SOX−1, Table 1 shows unadjusted *d* as it is below the minimum adopted for the measurement with SEM. In the a-SOX substrate case, the dislocation densities (*D*) were assessed with a fitting procedure—see Appendix A—of the XRD rocking curves, shown in Figure 1. In the KPI substrate case, we employed TEM for independently assessing *w* and estimating *D*. The HRSEM images of two films, one grown on the a-SOX and another on the KPI substrate, are shown in Figure 2 and Figure 3, respectively.

**Figure 4:**
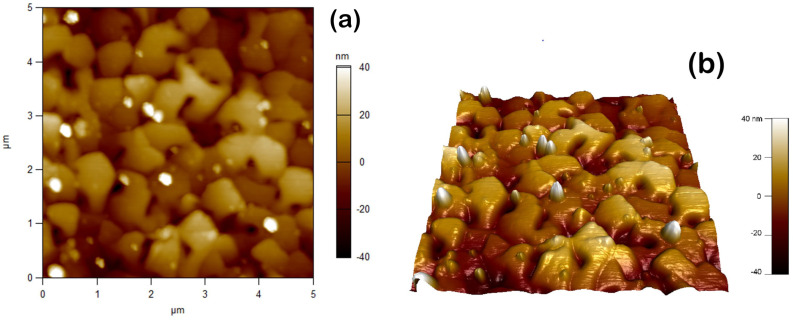
AFM images of the specimen KPI−5 listed in Table 2: (**a**) top view; (**b**) 3D view. AFM scan area and rate: 5μm×5μm and 0.98 Hz, respectively.

**Figure 5:**
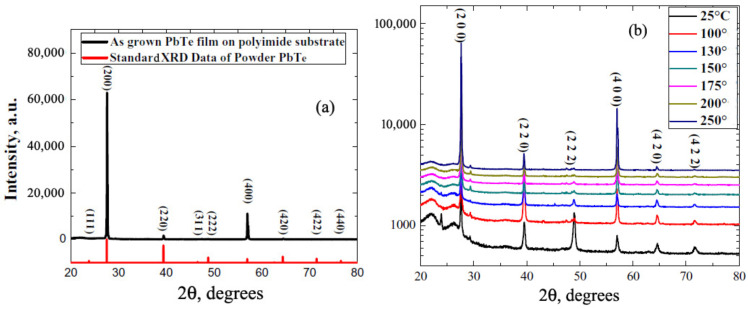
XRD spectra of the PbTe〈In〉 films on KPI: (**a**) the spectrum of specimen KPI−4 in Table 2; (**b**) the spectrum evolution with increasing the substrate temperature (inset—selected values of $T_{s}$ in the Celsius degrees).

**Figure 6:**
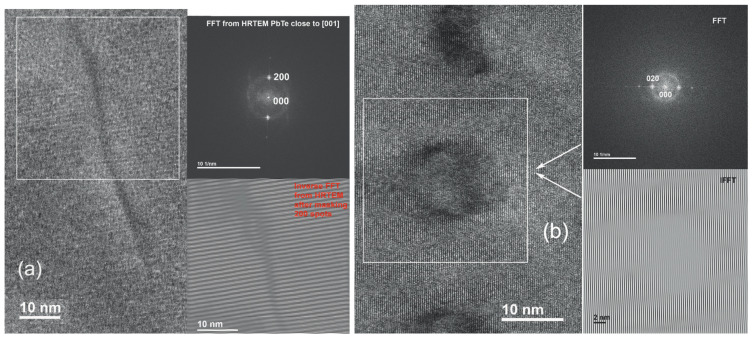
HRTEM images through 0.1μm thick lamina cut from a [001] PbTe〈In〉 film on KPI. Left and right sides—real-space and FFT (upper) and IFFT (lower) images, respectively. Obtained by a mask-assisted picking out of the diffracted electron beam FFT spots: (**a**) 200; (**b**) 020.

**Figure 7:**
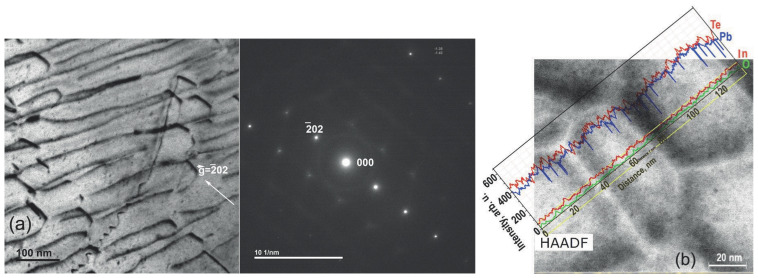
(**a**) HRTEM image over an area of ∼0.5μm×0.5μm through 0.1μm thick lamina cut from a [111] PbTe〈In〉 film on KPI. Obtained by picking out the spot $\bar{2}02$; (**b**) HAADF graph showing the EDXS results spatially, over an area of ∼115nm×115nm, on the STEM image background.

Figure 4 displays the AFM image of a film on the KPI substrate, which is specified in the caption of the figure. Figure 5 shows the XRD θ:2θ scan spectrum of some film grown on the KPI substrate, and the spectrum evolution vs. variation in $T_{s}$; see in the Figure 5a and Figure 5b, respectively. TEM images, disclosing subtle structural details of the films, are presented in Figure 6 and Figure 7. The HAADF graph in Figure 7b, given in units of signal intensity, was corrected for the background and tested laminae thickness noted above. The structural data will be discussed in detail in Section 4.1.

### 3.2. Charge-Carrier-Related Properties

The measurements described in Section 2.2 show that, for both substrates, PbTe〈In〉 films grown at $T_{s}>200$ °C have a conductivity of n-type, i.e., $R_{H}$ and *S* are both negative. The electron Hall concentration and mobility were obtained by the well-known relations, $n_{H}=1/\left(R_{H}e\right)$, e<0 is the charge of the electron, and $\mu_{H}=\left|R_{H}\right|\sigma$, respectively. These properties are shown in Table 3 and Table 4 below, will be discussed in Section 4.2.

### 3.3. Infrared Transmission Spectra

The SOI wafer, a-SiO_2_/Si(100)/a-SiO_2_, whose top was the film deposition substrate, served also as a test specimen for the measurement setup and simulation.

**Figure 8:**
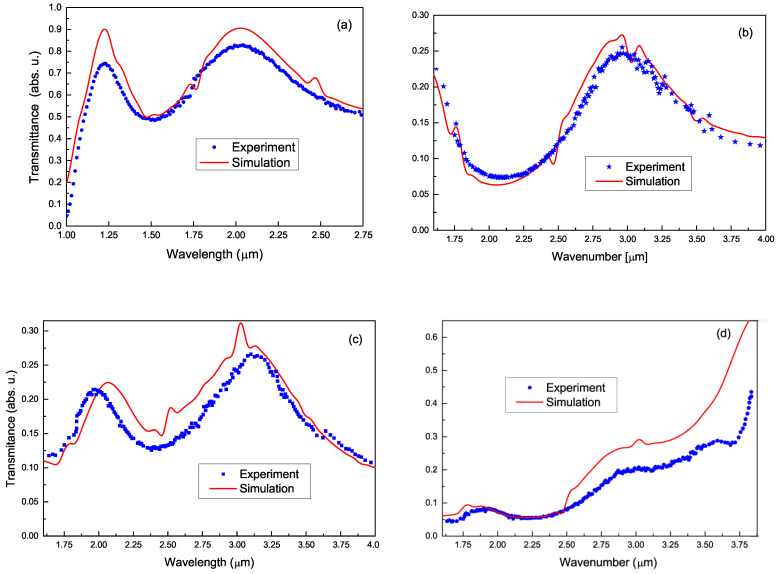
Spectra of IR transmission through studied multilayer structures: (**a**) SOI wafer; (**b**), (**c**), and (**d**) samples SOX−1, SOX−2, and SOX−3, respectively. Symbols—measured; full lines—simulated.

Four transmittance spectra, measured and simulated with the method developed in Appendix B and outlined in Section 2.3, including one via the SOI wafer and three via the PbTe〈In〉 films on this substrate, are shown in Figure 8 for the λ-ranges above and below the fundamental absorption onsets of Si, λ>1.1μm [Figure 8a] and PbTe, λ<4.0μm [Figure 8b–d], respectively. A detailed discussion of these results is presented in Section 4.3.

## 4. Discussion

### 4.1. Morphology, Texture, and Surface Roughness

Structural properties of a deposited polycrystalline film, such as morphology, texture, and surface roughness, are defined by used materials, growth technique, and growth conditions. The latter includes, in particular, the lattice, mechanical, and thermal compliance between the materials of the film and the deposition substrate. Variations in the structural properties are defined by processing conditions at which the grain nucleation, growth, coarsening, coalescence, and thickening occur. A lattice parameter mismatch between the substrate and deposited film’s materials, if any, results in strains that can be relaxed by a network of dislocations lying in the film–substrate interface and grain boundaries (GBs). The situation changes if there is also a mismatch in the thermal expansion coefficients, α. Under the latter mismatch, the strains build up stresses upon cooling samples from the growth or/and annealing temperatures to room one. When these stresses relax, they can break the film’s integrity by either delaminating or cracking the film, depending on the relation between the elastic properties of the film and substrate materials.

An outline of the formation of a polycrystalline film due for review [83] is as follows. It starts with nucleating isolated grains on the substrate’s surface and proceeds as the nuclei grow, coarsen, and touch the neighboring ones. Further lateral growth leads to the grains’ coalescence, forming GBs and defining at least the initial grain structure characteristics. An eventual film morphology is determined by a synergy of matching the lattice parameters at GBs, geometrical shadowing effect, and controlled surface diffusion of atoms too. Good matching provides integrity to the film, whereas their mismatch can lead to voids/pores when more than two crystallites overlap. If GBs formed via island impingement are immobile, the grain structure arising from the nucleation, growth, and coalescence processes is retained at the base of the film. Next, film thickening occurs through epitaxial growth just on these grains. Some authors suggest that columnar grains grow when the substrate is rough so its bumps may receive more atoms than valleys from all directions due to the above shadowing.

PbTe and a-SOX have essential mismatches in atomic arrangements and α. While a-SOX has no crystal lattice, a short-range order with a correlation length of ≳5Å persists in this material [84]. This value is close to the larger lattice constant of quartz c=5.405Å but appreciably smaller than the lattice constant of PbTe, cited in Appendix A. Also, a stable plateau value of $\alpha_{\mathrm{PbTe}}=2.0\times 10^{-5}\;K^{-1}$ persists at T>200K [3], whereas $\alpha_{{\mathrm{SiO}}_{2}}≲7.5\times 10^{-7}\;K^{-1}$ [85]. These mismatches in conjunction with the elastic modules’ closeness for PbTe and a-SOX, dictate the existence of a range for $T_{s}$, where a part of the films’ integrity cannot be kept when increasing *d* as described in Section 2.1. Our tries revealed 250 °C—see in the caption of Table 1—and −50 °C to be optimal values of the upper and lower bounds of such a range. Depositing these films at −50 °C $\leq T_{s}\leq 250$ °C allowed us, per prescribed *d*’s, to achieve the largest possible *w*, but revealed the growth to d>1μm to be unfeasible, as seen, e.g., in Table 1. The micron thickness limit is attainable at $T_{s}$ in the above range, though diminishing $T_{s}$ results in a decrease in *w*, which becomes drastic at $T_{s}<0$ °C. For example, a film grown at $T_{s}=-50$ °C to a micron thickness has grains with w=135nm; see Figure 2. These trends agree with those reported in an independent study [54] devoted to the deposition of PbTe films on a similar substrate.

For successful film deposition on the KPI substrate, matching the atomic arrangements of the film material and KPI makes no sense since KPI is a fully amorphous polymer, i.e., it possesses no even short-range inter-molecular order. Further, $\alpha_{\mathrm{KPI}}≃\alpha_{\mathrm{PbTe}}$, i.e., KPI and PbTe match excellently with regard to the thermal expansion. On the other hand, contrary to a-SOX, KPI has a high elastic mismatch with PbTe, viz., Young’s modulus of 100μm thick KPI is 3.6GPa [82], and that of crystalline PbTe is more than an order of magnitude larger. Such a difference is crucial for preventing micro-cracking of the grown PbTe〈In〉 films in the cooling processes, which may occur as outlined above. Thus, KPI seems well appropriate for depositing the films in question, which is confirmed by comparing the data in Table 1 and Table 2. Namely, the deposition on this substrate yields, at x=0.5at.%, as-grown PbTe〈In〉 films with *d* and *w* beyond those of their a-SOX substrate counterparts annealed. Also, as seen from Table 2, given *d* and *x*, the greater the $T_{s}$, the larger the *w*, while annealing an as-grown film increases, though not essentially, *w*; compare samples KPI−2 and KPI−3.

The structural evolution of the PbTe〈In〉 films follows, at large, the general trends in terms of the conditions and fundamental kinetic processes detailed in ref. [83], except for coalescence events, which are very rare, if any, due to our observations. Figure 2 and Figure 3 demonstrate good integrity and a tightly packed, practically not tilted, columnar structure of the films on both substrates used. Since both our substrates are highly smooth, these figures allow us to conclude with caution that surface roughness may not be a prerequisite for columnar-type film growth. A recent paper [86] on preparing films of a similar grain structure on a substrate like a-SOX, but with a quite different compound and deposition method, convinces us that it is the substrate amorphousness that is most decisive for achieving such a morphology.

Concerning *D* for the a-SOX substrate, it decreases with increasing *d* as is expected, but this decrease is slow. Indeed, as seen in Table 1, upon increasing *d* by factor ∼8.2, *D* decreases by a factor ∼1.9 only, but remains at the $10^{11}\;{\mathrm{cm}}^{-2}$ order of magnitude. These peculiarities are in marked contrast with the dislocations’ characteristics typical for the PbTe films grown with the epitaxy methods, such as, e.g., MBE [36] and references therein, HWE [87,88,89], and modified HWE [90]. For example, in the MBE-grown PbTe films, D(d) rapidly decreases about inversely to *d*, from $D\left(0.1\;\mu m\right)≃10^{9}\;{\mathrm{cm}}^{-2}$ down to $D\left(1.0\;\mu m\right)≃10^{8}\;{\mathrm{cm}}^{-2}$. So far, to our knowledge, no systematic study on the dependence of *D* on *d* for the HWE-grown PbTe has been published. Yet, for a few such films on the a-SOX substrate with an unspecified *d*, fragmentary data of much smaller D≃(0.87–$4.7)\times 10^{5}\;{\mathrm{cm}}^{-2}$ were reported [90]. Let us note that, with such large *D*’s as we assessed in this case, the dislocations were shown [24] to localize phonons near them. Analysis of scans shown in Figure 4 allows for assessing the RMS surface roughness for the film KPI−5 as being 25±5nm, much smaller than its *w* shown in Table 2. As is seen from Figure 4, tiny apexes and pits, overshooting the RMS roughness level, are extremely rare.

In materials science, the texture of a polycrystal means the distribution of crystallographic orientations of its grains. A sample in which these are fully random is stated to have no distinct texture. When few allowed orientations are observed, the film is said to have a weak, moderate, or strong texture subject to the percentages of detectable grain orientations. As Figure 5a shows, the film grown on the KPI substrate at $T_{s}=250$ °C has a strong (200) texture. Figure 5b illustrates how this texture evolves upon increasing $T_{c}$, from the moderate texture at $T_{s}=25$ °C via similar ones, all containing additional XRD reflexes. These peaks, being orders of magnitude smaller than the main one at all $T_{s}$, drop further to being negligible at $T_{s}=250$ °C, which proves a good grain crystallinity of the films obtained at the optimized fabrication regime. In addition, XRD peaks typical of Pb, Te, and PbO phases were not found, indicating that, within the accuracy of the XRD instruments employed, the films are single-phase. Also, there are samples at our disposal grown with nearly [111] surface orientation, whose XRD spectra displayed at an optimal $T_{s}$ the finally strong $\left(\bar{2}02\right)$ texture.

Usual XRD spectra, such as in Figure 5, cannot reveal fine structural imperfection features, extra phases, and low-concentration impurities, which remain even after optimizing the film synthesis. For these purposes, we used the TEM imaging presented by Figure 6 and Figure 7. One can see the misfit defects and minute extra plane in sub-figures (a) and (b), respectively, of Figure 6. The dislocations and defects create stresses that appear as linear features or rounded ones, as seen inside highlighted squares in Figure 6. Based on the scale shown in Figure 6, it can be concluded that the spatial dimensions of the defects are much smaller than the grain sizes, while not every grain contains defects. The inverse FFT (IFFT) images in Figure 6 ascertain that the grain is well crystalline beyond the defect.

A dislocation network in real-space HRTEM images forms cells of various shapes, depending on the orientation of the grains, which is seen in Figure 7a on the left. The dark contrasts there are due to stresses developed in compliance with the grains’ structure and orientation. Applying the line intercept method—see in Appendix A—to Figure 7a with manual intercept counting, we obtained a rough estimate of D∼$10^{9}\;{\mathrm{cm}}^{-2}$. The STEM image in Figure 7b shows the surface topography of the lamina itemized in Figure 7a with the HAADF graph of the spatial content distribution of elements detected with EDXS. It is seen that, except for the host compound (Pb, Te) and doping (In) atoms, a small content of oxygen (O) is found, which may be due to oxidized-lead-phase precipitates at GBs. The imbalance between Pb and Te in favor of Te seen in the graph is worth noting, which is integrally weak but locally, at relatively rare dips, strong. This is highly likely a combined impact of non-stoichiometry (Pb vacancies) and the substitution of Pb with In. The HAADF graph seemingly indicates a spatial correlation between the distributions of dislocations and Te ions.

### 4.2. Electron Density and Transport Properties

The common trend when depositing the PbTe films using evaporation techniques is eventual non-stoichiometry. To compensate for losses of Pb, Te, or both in the epitaxy methods [36,87,88,89,90], either an intricate Pb-Te source vapor-pressure management is to be undertaken, or additional Pb or/and Te vapor source/s placed underneath the main one are to be added. When synthesizing the films, the addition of In into the evaporation source at a content *x*, below its solubility limit of 5at.%, facilitates alleviating strict stoichiometry control since the In impurities fill a part of emergent Pb vacancies (Pb substitution), thus decreasing the non-stoichiometry and improving the crystal lattice integrity. Physical effects of this impurity, and other group-III ones, in single-crystalline IV-VI semiconductors were extensively studied and summarized in early reviews [79,80] and in a later one [78]. It is revealed [78,79,80] that, in these crystals, the In dopants are donors, but $n_{H}$ does not increase with *x*; rather, it stabilizes at a level not exceeding several units of $10^{18}\;{\mathrm{cm}}^{-3}$ while increasing *x* from 0.3 to 2.5at.% over the temperature range of T=4.2–300K.

The $n_{H}$ data displayed in Table 3 and Table 4 show that this is the case also for the PbTe〈In〉 nanocrystalline films grown on both used substrates. This is one more independent proof of a tightly packed crystalline grain structure of the films in question. The actual electron concentration *n* relates to $n_{H}$ by $n=n_{H}/r_{H}$, where $r_{H}$ is the Hall factor. Calculation due to an appropriate band structure model ([77] Chapter 5) for the PbTe case yields $r_{H}≃0.816$, i.e., *n* is only slightly larger than $n_{H}$. That $n\ll N_{\mathrm{In}}$, where $N_{\mathrm{In}}=x/v$ is the In density, in which *v* is the primitive cell volume, is not explainable by seemingly naturally assuming that each In substitutes a Pb^+2^ ion, and resides there in the In^+3^ valence state, i.e., donates an electron to the conduction band.

The current explanation—see, e.g., [78]—involves two fairly independent effects such as self-compensation and chemical potential (Fermi-level) pinning due to the appearance in the conduction band of quasi-local resonant In-impurity levels. In the present case, the former implies that the In-doping stimulates the formation of defects with the opposite, i.e., acceptor electrical activity, such as a vacancy or In^+1^ in the Pb site, or electrically inactive In^+2^ in the Pb site and interstitial In^0^. Thus, the In-donor ability in PbTe proves suppressed to a large extent, so *n* in self-compensated samples becomes sufficiently small. This is why we notate In-doped PbTe due to [78,79,80], and not as a substitution alloy. However, such a property of PbTe〈In〉 as its unique spatial homogeneity of *n*, and the physical effects resulting from it [78,79,80], can be understood only by invoking Fermi-level stabilization that acts alone or in synergy with the self-compensation.

The data on $n_{H}$, assessed as described in the text, and measured values of *S*, shown in Table 3 and Table 4, allow for the extraction of Fermi-level energy provided that the theory for doped crystalline PbTe [3,5], ([77] Chapter 5), and n-type PbTe〈In〉 [78,79,80] is also applicable to the films under study. This assumption seems rather reasonable since $n_{H}$ is practically the same as in the PbTe〈In〉 bulk source [53], and $R_{H}$ and thus $n_{H}$ are practically constant vs. *T* over the range of 80–300K as in the PbTe〈In〉 single crystals [3]. As seen from Table 3 and Table 4 and their comparison, *S* shows minute variation with fabrication conditions and dimensional parameters of the films, while it decreases at increasing *n* due to theory, being comparable to *S* of bulk PbTe〈In〉 [3]. This supports the Fermi-level stabilization [78,79,80] in the grains. To further approbate this idea, it would be expedient to compare the values of *S* presented in this article with those reported in previous related studies shown in Table 5.

Comparing the data on *S* in Table 5 with those in Table 3 and Table 4 shows that our *S* are well close to the reference ones given comparable *n*, irrespective of the synthesis method. The larger the *n*, the smaller the |S|—see lines no. 4 and 7 in Table 5—as should be the case and also is for our data.

In contrast, $\mu_{H}$ shows high variability with those parameters since mobility critically involves electron scattering mechanisms. First of all, the values of $\mu_{H}\left(300\;K\right)$ for both substrates are about twice as small as that for bulk crystals [3,5] and relatively slightly lower than for epitaxial films of n-PbTe with comparable *n* [36,89]. Like in those, $\mu_{H}\left(T\right)$ increases with decreasing *T* due to weakening the electron–phonon scattering, being the main mechanism limiting the mobility in crystals and crystalline films of LCs [3,5,36], and this divergence in $\mu_{H}\left(T\right)$ increases, becoming notably stronger at lower *T* inquired. Though, as seen by comparison of $\mu_{H}\left(T\right)$ for samples KPI−2 and KPI−3, the full-cycle heat treatment strongly increases mobility, which becomes strikingly high at T=10K, and the above deviation persists even in this best case. The reason for that is the developing dominance of electron scattering by structural defects such as GBs and, possibly, dislocations. An essential role that dislocations may play can be concluded from comparing the data on $\mu_{H}\left(80\;K\right)$ in Table 3 and Table 4. Indeed, for the last three films on KPI, $\mu_{H}$ prove much larger than those for all films on a-SOX, which can be explained by two orders of magnitude larger *D* in the latter films. The resonant scattering of electrons by In-impurity centers can also contribute to limiting mobility but, to our knowledge, this problem is still unexplored.

The above noted procedure of calculating the Fermi energy vs. *T* concurrently using $n_{H}$, largely unaffected by defects S(T) dependence, allows for calculating the mobility of a hypothetical ‘defectless’ film. As we have reported [31,57], combining this and experimental $\mu_{H}\left(T\right)$ dependences makes it possible to extract the contribution of defects to the conductivity of the real fabricated films. Detailed exposition of this approach, however, is beyond the scope of this article.

### 4.3. Transmission Spectra

Now, consider Figure 8, to discuss to what extent the measured spectra can fit the rigorous EM-optical model, developed in Appendix B, with subsequent smoothing of the fringes due to the recipe of Appendix B.3. We are to state in advance that no accurate agreement between the theory and experiment would be expected as the commercial SOI wafer does not satisfy a condition at which the above-noted theory would be wholly valid provided that physical parameters are accurately known. This is the optical smoothness of all interfaces, whereas the a-SOX layer at the SOI wafer backside is rough. Thus, it is this roughness that, due to [91], might be a main reason for the discrepancy between the theory and experiment, seen in Figure 8a.

Yet, as the above figure shows, despite the amplitude misfits, the measured data and simulated curves are well congruent to each other, and display interference extrema at the same λ of ∼1.2μm,2.0μm for peaks and ∼1.51μm for dips, while, apart from these, the agreement is fairly good. This indicates that the nominal thicknesses of the SOI wafer sub-layers and their complex RIs plugged into this simulation were highly likely accurate. This conclusion cannot be regarded as quite reliable in the range near the onset of fundamental absorption in Si. Here, due to much uncertainty existing in the compiled data for the complex RI of Si, even a small inaccuracy in the Si slab thickness could result in an appreciable change in the simulated transmittance contributing to its discrepancy with the measured counterpart; see Figure 8a. One more cause for the above disagreement would be a diffuse scattering of light by point defects in the Si slab, the treatment of which is not reducible to the plain procedure suggested in Appendix B.3.

The spectra of the PbTe〈In〉 films shown in Figure 8, like those of their substrate, have an oscillation structure: the thinner the PbTe film, the larger the oscillation amplitude and the wave-number frequency, while, for sample SOX−3, the oscillation is suppressed above λ≃3.1μm. The spectrum of the thickest film SOX−4 shows no oscillation. Since, in the considered spectral range, PbTe absorbs the radiation, the influence of the backside a-SOX roughness is expected to weaken, while effects of structural defects would emerge. For these films, there are the following extra causes of discrepancy between the theoretical and experimental transmittance: (i) diffuse scattering at the interfaces of PbTe〈In〉 layer with an a-SOX one and air and structural defects such as GBs and dislocations; (ii) inaccurate *d*, and n+iκ of the PbTe〈In〉 layer. In this context, the inaccuracy of *d* means {not only the error in measuring its average, shown in Table 1 and Table 2, but also involves local variations around the average along the film surface. These variations may likely occur as the area tested by HRSEM is notably smaller than the irradiation area in transmission measurement.

Figure 8b shows a fair agreement between theory and experiment since their deviation is of only about −1% at the dip and +3% at the peak at worst. In Figure 8c, the agreement is less good but quite reasonable. At λ≥3.1μm, the theoretical curve and the data graph deviate to within ±1%, while, at 2.1μm<λ<3.1μm, they are more or less congruent with the worst deviation of 3% if disregarding the bump at ∼3.0μm. An appreciable discrepancy occurs at λ<2.1μm, where the lower oscillation peak in the simulated spectrum is red-shifted relative to its measured counterpart. As seen from Figure 8d, for sample SOX−3 a perfect agreement, to within less than 0.3%, is observed over the short-wavelength range of 1.6μm<λ<2.4μm and, above this range, the discrepancy rapidly grows. For sample SOX−4, the simulated and measured data are overall discrepant.

The above spectra properties can be treated as follows. A fair agreement for sample SOX−1 means that the above factors (i) and (ii) have no strong effect on the spectrum of sample SOX−1, in particular, that this film has no appreciable lateral variations in *d* from its nominal value in Table 1, and has high integrity. In sample SOX−2, the diffuse scattering, factor (i), likely has a mild effect, but the *d* and complex-RI inaccuracy, factor (ii), essentially affects the transmission. For sample SOX−3, both factors at λ≥2.4μm become essential to account for the observed discrepancy. Indeed, the complex RI of a given PbTe〈In〉 film may effectively differ from the tabular one of crystalline PbTe that we used in simulations due to strains created by high-density dislocations—see Table 1—and voids, even minute, between GBs. Such a medium effect known for polycrystalline Si films for a long time should contribute to the small and mild spectra discrepancies for samples SOX−1, SOX−3 at 1.6μm<λ<2.4μm, and SOX−2, respectively. So far, to our knowledge, no practically usable theory of the diffuse scattering effect on the transmission exists. For a discussion on the issue in the wafer backside roughness case, see [91].

## 5. Conclusions

The development in the field of research on LTCs, PbTe in particular, has experienced renewed, steadily growing interest in the last two decades. This article links the thorough study on improvement of the PVD synthesis of the PbTe〈In〉 films and their characterization in the main text, a mini-review of studies in the above research field for seven decades in the introduction, two methods for the analysis of dislocations in Appendix A, and an original unpublished method of simulating an optical multilayer in Appendix B.

Two substrates—a-SOX (atop the SOI wafer) and KPI—were employed for the film deposition. These sections include renewing and tuning the deposition regimes concerning the film composition and arrangement of crystallites that are optimal for having the best structural properties, such as dominant texture, integrity, and small surface roughness. The prepared films were characterized structurally with a wide range of techniques, which showed a closely packed columnar structure, dominant (100) or (111) textures, i.e., a good grain crystallinity, and sizes from tens and hundreds of nanometers for the a-SOX and KPI substrates, respectively. Physical and technological grounds that favor the feasibility of deposition on these substrates are amply considered in Section 4.1. Though amorphous, a-SOX supports a short-range order whose scale and the lattice parameter of PbTe notably mismatch, which limits the PbTe〈In〉 films grown on a-SOX to be nanocrystalline and nano-scale thick. At the same time, the complete amorphousness of KPI allows for the deposition of the films in question, which vary from nano- to microcrystalline, with notably larger *d* up to 4μm; compare the data in Table 1 and Table 2. This, if remaining in the realm of thin films, points at no limitation compared to substrates, mentioned in the introduction and well approbated in research labs but proving mostly impractical for use in applications. Beyond the amorphousness of the substrates used in our study, allowing one to grow on them the PbTe〈In〉 films with the above highly demanded structural properties, a key advantage of them over others, applicable for that deposition, is fully compatible with integrated circuits technology (both); SOI technology (a-SOX); flexible printed electronics (KPI), to mention a few. Both a-SOX and KPI are resistant to high temperatures, and KPI to many chemicals. Also, the structural limitations and high *D* inherent to the films grown on a-SOX invert to advantages when using these films for TE applications [7,8,10,12,13,15,16,22,23,24,25,26,28,31].

Using the results of electrical and TE transport characterization by the σ, $R_{H}$, and *S* measurements and calculations, we reliably confirmed that adding In vapor to the PbTe PVD process stabilizes Fermi-level energy and electron concentration over the grains in the PbTe〈In〉 films. A semi-phenomenological numerical procedure, which allows one to extract from experimental $\mu_{H}$ a contribution of scattering of electrons by structural defects, has been reported by us elsewhere [31,57]. Combining the results of structural and transport properties analyses allows for casting out the worst and selecting the best samples, thus rendering feedback for optimizing the films’ synthesis process.

The use of IR transmission for the structural characterization of thin films is rare in the present field. It has been mostly used for experimental determination of either RI when a film, typically bi-layer, is cut with precisely known thicknesses or the thicknesses when RI is exactly known [92]. In this study, we realized a fundamentally new approach that facilitates separating more-or-less structurally perfect PbTe〈In〉 films, such as SOX−1 and SOX−2, from imperfect ones, such as samples SOX−3 and SOX−4. At the same time, they all are not particularly distinguished from the viewpoint of electron and atomic force microscopy, as well as $\mu_{H}$ and *S*. The PbTe〈In〉 films with good grain crystallinity, well-controlled morphology, and surface roughness reported in this study are highly prospective for various device applications. In the a-SOX substrate case, these may be TEGs and IR PCDs in the Si integrated circuits framework, while, for the KPI substrate, flexible and wearable IR PC sensors can be foreseen.

## Figures and Tables

**Table 1:** Structural parameters of selected PbTe〈In〉 films with In content x=0.5at.% grown on the a-SOX substrate at $T_{s}=250$ °C, and annealed as described in the text.

Specimen	Mean Film Thickness d,nm	Mean Lateral Grain Size *w*, nm	Dislocations Density $D,10^{11}{\mathrm{cm}}^{-2}$
SOX−1	90	180±27	1.7
SOX−2	165±9	190±28	1.1
SOX−3	295±15	200±30	0.9
SOX−4	741±37	200±30	0.9

**Table 2:** Structural parameters of selected PbTe〈In〉 films grown on the KPI substrate at various substrate temperatures as described in the text.

Specimen	In Content x,at.%	Substrate Temperature $T_{s}$, °C	Mean Film Thickness d,μm	Mean Lateral Grain Size *w*, nm
KPI−1		300	2 ± 0.10	180±30
KPI−2	0.5	350	3 ± 0.15	641±70
KPI−3 ^†^		350	3 ± 0.15	656±70
KPI−4		250	2 ± 0.10	114±11
KPI−5	1.0	325	3 ± 0.15	586±62
KPI−6		325	4 ± 0.20	640±69

^†^ KPI−3 was obtained from KPI−2 by subjecting it to full-cycle heat treatment as described in the text. The other samples presented in the table were studied as grown.

**Table 3:** Electron Hall concentration $n_{H}$ at T=80K, Hall mobility $\mu_{H}$ at T=80K and 300K, and thermopower *S* at T=300K, measured for the films presented in Table 1.

Specimen	$n_{H},\;10^{18}\;{\mathrm{cm}}^{-3}$	$\mu_{H}@300\;K$	${\mathrm{cm}}^{2}V^{-1}s^{-1}$	$\mu_{H}@80\;K$	S,μV/K
SOX−1	4.8	700		6500	−180
SOX−2	5.1	800		7200	−185
SOX−3	5.0	850		8000	−182
SOX−4	5.0	900		8800	−180

**Table 4:** $n_{H}$, $\mu_{H}$, and *S* of the films presented in Table 2. The Hall concentration and Seebeck coefficient are shown for T=300K, whereas the mobility is shown for that and the other two lower temperatures.

Specimen	$n_{H},\;10^{18}\;{\mathrm{cm}}^{-3}$		$\mu_{H},\;\;{\mathrm{cm}}^{2}V^{-1}s^{-1}$		S,μV/K
@T		300K	80K	10K	300K
KPI−1		–	–	–	−150
KPI−2	8.3	630	1900	1600	−160
KPI−3		910	8000	20,000	−160
KPI−4		500	950	725	−160
KPI−5	9.4	500	1050	800	−160
KPI−6		500	2050	1750	−150

**Table 5:** Published reference values of the Seebeck coefficient at 300K for n-type PbTe materials.

S,μV/K	Material	Synthesis	$n_{H},\;10^{18}\;{\mathrm{cm}}^{-3}$	Doping, at. %	Reference
−180			5.0	0.5,1	[11]
−200	PbTe〈In〉		4.8	0.05	
−191		Alloy casting	5.1	0.1	[28]
−92	PbTe〈I〉		14	0.1	
−175				1	
−187	PbTe〈In〉	PECS	2	0.1	[14]
−200				0.05	
−90	n-PbTe	Hot pressing	40		[15]
−100	n-PbTe	THM ingots			[13]

## Data Availability

The original contributions presented in the study are included in the article, further inquiries can be directed to the corresponding author, Zinovi Dashevsky, zdashev@bgu.ac.il.

## Abbreviations

The following abbreviations are used in this manuscript:

LC	Lead chalcogenide
LTC	Lead–tin chalcogenide
IR	Infrared
TE	Thermoelectric
TEG	TE generator
PD	Photon/photo detector
PC	Photo-conductive
PCD	PC detector
CBD	Chemical bath deposition
CVD	Chemical vapor deposition
PVD	Physical vapor deposition
RT	Room temperature
EBPVD	Electron-beam-assisted physical vapor deposition
KPI	Kapton^®^ polyimide
SOI	Silicon on insulator
SOX	Silicon dioxide
a-SOX	Amorphous SOX
XRD	X-ray diffraction
SEM	Scanning electron microscope/microscopy
HRSEM	High-resolution SEM
TEM	Transmission electron microscope/microscopy
STEM	Scanning TEM
HRTEM	High-resolution TEM
AFM	Atomic force microscope/microscopy
EDXS	Energy-dispersive X-ray spectroscopy
HAADF	High-angle annular dark field
MBE	Molecular-beam epitaxy
HWE	Hot-wall epitaxy

## Appendix A. Assessing the Dislocation Density

We estimated the dislocation density *D* using methods based on XRD [93] for the a-SOX substrate and on TEM imaging [94] for the KPI substrate cases. Due to [93], a measured XRD rocking curve width $\beta_{M}$ is given by

(A1) $$\beta_{M}^{2}=\beta_{0}^{2}+\beta_{\alpha }^{2}+\beta_{\varepsilon }^{2}+\beta_{L}^{2}+\beta_{R}^{2},$$

where $\beta_{0}$ is an instrumental half width and $\beta_{\alpha }$, $\beta_{\varepsilon }$, $\beta_{L}$ and $\beta_{R}$ are the widths due to lattice tilting, local strain, particle size, and uniform lattice bending, respectively. Assuming, as is customarily accepted, that $\beta_{L}<<\beta_{M},\;$ and $\beta_{R}<<\beta_{M}$, whereas, with our high-precision apparatus, $\beta_{0}$ is negligible compared to other contributions, Equation (A1) simplifies to the following equation:

(A2) $$\beta_{M}^{2}=\beta_{\alpha }^{2}+\beta_{\varepsilon }^{2}.$$

The parameters $\beta_{\alpha }$ and $\beta_{\varepsilon }$ can be related to some coefficients $K_{\alpha }$ and $K_{\epsilon }$, as well as to the Bragg angle θ, by $\beta_{\alpha }^{2}=K_{\alpha }$ and $\beta_{\varepsilon }^{2}=K_{\varepsilon }\tan^{2}\theta$. Upon this representation, Equation (A2) becomes

(A3) $$\beta_{M}^{2}=K_{\alpha }+K_{\varepsilon }\tan^{2}\theta .$$

On the other hand, one has $K_{\alpha }=\left(2\pi \ln2\right)b^{2}D,\;K_{\varepsilon }=\left(8\ln2\right)\bar{\varepsilon^{2}}$, where *b* is the modulus of Burgers’ vector $\vec{b}$, *D* is defined above, and $\bar{\varepsilon^{2}}$ is the mean-square strain in the normal direction to the diffraction plane [93]. The first of the above relations is plainly rewritten as

(A4) $$D=\frac{K_{\alpha }}{\left(2\pi \ln2\right)b^{2}}$$

which allows for a straightforward assessment of *D* once an estimate of $K_{\alpha }$ and knowledge of a specific $\vec{b}$ are at one’s disposal.

To the above end, the XRD rocking curves, for several (h00) reflections, here, (200),(400), and (600), were measured and analyzed. For the analysis, we assumed, as usual, each of those curves to be of Gaussian shape and, this way, extracted its full width at half maximum (FWHM) at the Bragg angle θ.

On the basis of Equation (A3), the values of $\beta_{M}^{2}$, which differ from the thus-extracted FWHMs squared only by a known constant factor, were fitted to a linear function of $\tan^{2}\theta$. These fits yielded the lines’ slopes $K_{\varepsilon }$ and, upon the back extrapolation, the values $K_{\alpha }$ of their intercepts with the ordinate line $\tan^{2}\theta =0$. In addition, we used Burgers’ vector $\vec{b}=\frac{a}{2}\langle 110\rangle$, typical of the FCC crystal structure compounds with the lattice constant *a*, equal to 6.461Å for PbTe. Finally, substituting the $K_{\alpha }$ thus obtained and modulus of the quoted $\vec{b}$ into Equation (A4), we calculated the sought *D* estimates shown in Table 1.

Alternatively, *D* can be assessed with the line intercept method, widely applied in the cases used, where $D<10^{14}\;{\mathrm{cm}}^{-2}$ [94]. Knowing the thickness *t* of the lamina subjected to TEM, the intersection count *N* between a given grid and the dislocations on a STEM image, such as, e.g., shown in Figure 7a, and the total length of the grid *l*, one can estimate the dislocation density by D=2N/(tl).

## Appendix B. Modeling the Optical Properties of Planar Multilayered Structures

### Appendix B.1. Outline

By the planar multilayered structure (multilayer for short), we mean, as commonly accepted ([95] Chapter 1), ([96] Chapter 4), ([97] Chapter 2), a stack of layers with plane-parallel boundaries made, in general, of different materials. We choose the right-hand coordinate frame, in which the in-plane (lateral) and out-of-plane (in-depth) coordinates are (x,y) and *z*, respectively. Let the upper and lower interfaces of the multilayer with input and output semi-space media be the planes $z=z_{0}=0$ and $z=z_{L}<0$, respectively, where *L* is the number of the layers, and any finite layer with a number, say, *i*, occupies the space $z_{i-1}\leq z\leq z_{i}$ (1≤i≤L), where the interfaces’ coordinates are negative, decrease, and arbitrary in any other respect. In advance, we consider only layers perfect in volume and bonding, with optically smooth interfaces, so only first-principle EM calculations are performed. An in-house developed method for calculating the optical properties of such an ideal multilayer, but arbitrary in terms of the layers’ number and their thicknesses, is described in the next Appendix B.2. The optical interference in the multilayer can be broken down due to several causes if it submerges layers with a thickness hugely larger than the wavelength of the impinging radiation. These causes and some heuristic post-processing of the calculated optical properties to mimic their effect within the rigorous electromagnetics framework are described further in Appendix B.3.

### Appendix B.2. Mathematical Method

Consider a monochromatic, linearly polarized, plane EM wave with a wavelength λ, amplitude $E_{0}$, and polarization vector $\vec{e}$, impinging the multilayer at an angle ϑ in the incidence plane x=0, from the semi-space z>0. The electric field vector phasor of the wave reads

$${\vec{E}}_{\mathrm{in},\omega }\left(\vec{r}\right)=\vec{e}E_{0}e^{j{\vec{k}}_{\mathrm{in}}\cdot \vec{r}};\;{\vec{k}}_{i}=k_{0}\left(\sin\vartheta ,0,-\cos\vartheta \right),\;\vec{r}=\left(x,y,z\right),\;k_{0}=2\pi /\lambda ,$$

where $\omega =ck_{0}$ is the circular radiation frequency and $j=\sqrt{-1}$ is the imaginary unit.

For the s-polarization, $\vec{e}=\left(0,1,0\right)$, so the electric field phasor vector has only one, *y*, component, which we call on this occasion *wavefunction* and denote as $\Psi (\vec{r})$, while omitting the subscript ω. It appears that $\Psi \left(\vec{r}\right)=E_{0}e^{ik_{0}x\sin\vartheta }\psi \left(z\right)$, where the piece-wise functions ψ(z), automatically satisfying Maxwell’s equations, for the reflection from, transmission via the multilayer to the semi-space $z<z_{L}$, and interior of an *i*-th layer, are given by

(A5) $$\psi_{0}\left(z\right)=r_{0}e^{jk_{0}q_{0}z},\;\psi_{L+1}\left(z\right)=t_{L+1}e^{-jk_{0}q_{L+1}\left(z-z_{L}\right)},$$

and

(A6) $$\psi_{i}\left(z\right)=r_{i}e^{jk_{0}q_{i}\left(z-z_{i-1}\right)}+t_{i}e^{-jk_{0}q_{i}\left(z-z_{i-1}\right)}\;\;\left(i=1,2,\ldots ,L-1,L\right),$$

respectively. For 0≤i≤L+1 above, $q_{i}=\sqrt{\epsilon_{i}-{\left(\sin\vartheta \right)}^{2}}$, where $\epsilon_{i}$ is the optical permittivity of an *i*-th domain. By definition, $\sqrt{\epsilon_{i}}=n_{i}+j\kappa_{i}$, where $n_{i}$ and $\kappa_{i}$ are the optical constants, i.e., refractive index and extinction coefficient, respectively. For the p-polarization, however, $\vec{e}=\left(\cos\vartheta ,1,\sin\vartheta \right)$, so the electric field phasor vector has two, namely, *x* and *z*, components. In this case, it is more convenient to use the magnetic field vector phasor ${\vec{H}}_{\mathrm{in},\omega }\left(\vec{r}\right)$, which, like ${\vec{E}}_{\mathrm{in},\omega }\left(\vec{r}\right)$ in the s-polarization case, has only a *y* component. Thus, it can also be reduced to another wave function $\Psi \left(\vec{r}\right)=H_{0}e^{ik_{0}x\sin\vartheta }\psi \left(z\right)$, where $H_{0}$ is the incident magnetic field amplitude and ψ(z) obeys the *same* relations as shown in Equations (A5) and (A6).

Finally, the unknown amplitudes $r_{i}$ and $t_{i}$ are to be determined with Equations (A5) and (A6). To this end, one should apply to the multilayer’s interfaces the EM boundary conditions ([95] Chapter 1) along with the requirements of normalized-transmission input, $t_{0}=1$, and zero-reflection output, $r_{L+1}=0$. This yields a system of 2(L+1) equations for the 2(L+1) amplitudes ${\left\{r_{i}\right\}}_{0}^{L}$ and ${\left\{t_{i}\right\}}_{1}^{L+1}$, in which these variables are essentially pairwise coupled. A standard approach to solving such a system of equations is using the method of Abeles’ 2×2 characteristic matrix (M-matrix) ([95] Chapter 1). While, in the past, some authors dubbed it as the matrix method—see, e.g., ([96] Chapter 4)—these days, the term transfer matrix (T-matrix) method—see, e.g., ([97] Chapter 2)—is more often used.

We consider quite a different technique based on the scattering theory ansatz, i.e., $r_{i}=s_{i}t_{i}$, which leads to an effective decoupling of $r_{i}$ and $t_{i}$, namely, to separate systems of equations for each of these arrays. AU: It’s OK The first, nonlinear system can be solved by backward iterations starting, due to $r_{L+1}=0$ and $t_{L+1}\neq 0$, with $s_{L+1}=0$ as follows:

(A7) $$s_{i}=\frac{\rho_{i,i+1}+s_{i+1}}{1+\rho_{i,i+1}s_{i+1}}e^{2j\delta_{i}},\;\rho_{i,i+1}=\frac{{\bar{q}}_{i}-{\bar{q}}_{i+1}}{{\bar{q}}_{i}+{\bar{q}}_{i+1}},\;\delta_{i}=\frac{2\pi d_{i}q_{i}}{\lambda }\;\;\left(i=L,L-1,\ldots ,1,0\right).$$

Here, $d_{0}\equiv 0$, and $d_{i}=z_{i-1}-z_{i}$ is the thickness of the *i*-th layer if 1≤i≤L. The expression of ${\bar{q}}_{i}$ equals $q_{i}$ or $q_{i}/\epsilon_{i}$ depending on whether the s- or p-polarization is used, respectively, and the difference arises due to a distinction in the corresponding boundary conditions. The second system of equations, in which $s_{i}$ plays the role of external parameters, turns out to be a *linear* system. In fact, when the whole array ${\left\{s_{i}\right\}}_{0}^{L}$ is already computed, the system in question can be solved by forward linear iterations starting with $t_{0}=1$ as follows:

(A8) $$t_{i+1}=\frac{\tau_{i+1,i}}{1+\rho_{i,i+1}s_{i+1}}e^{j\delta_{i}}t_{i},\;\tau_{i+1,i}=\frac{2{\bar{q}}_{i}}{{\bar{q}}_{i}+{\bar{q}}_{i+1}}\;\;\left(i=0,1,\ldots ,L-1,L\right).$$

After accomplishing the iterations given by Equation (A7), one can proceed to the iterations given by Equation (A8), and at once compute the multilayer’s reflectivity (reflectance) R. In the end, when finishing the iterations given by Equation (A8), the multilayer’s transmittance T can readily be calculated. These final results read as

(A9) T=Req¯L+1q¯0tL+12,R=s02,

where the real part would make sense if $\epsilon_{L+1}$ is a complex number, i.e., in the case of absorbing output, while ${\bar{q}}_{0}$ is real since $\epsilon_{0}$ is such by default. When the output and input media are the same, the fraction factor of T in Equation (A9) clearly has unity, since $\epsilon_{0}=\epsilon_{L+1}\Rightarrow {\bar{q}}_{L+1}={\bar{q}}_{0}$. In addition, at ϑ=0, i.e., at normal incidence, R and T do not depend on the polarization state, so, in this case, one also can use Equations (A5)–(A9) if the incident radiation is non-polarized but still monochromatic.

### Appendix B.3. Effects of Super-Wavelength-Thick Layers

To simplify the discussion, assume that ϑ=0 and all layers weakly absorbing, viz., $\kappa_{i}\ll n_{i}$. If all the layers are sub-wavelength-thick ones, i.e., satisfy $n_{i}d_{i}≲\lambda$, then the R and T vs. λ (or wavenumber $\lambda^{-1}$) curves are smooth. But, if there emerges at least one moderately super-wavelength-thick layer, which satisfies $n_{i}d_{i}>\lambda$, these curves start to oscillate. These oscillations, if even few, called interference *fringes*, are very close to periodic in $\lambda^{-1}$, but not strictly such unless $\kappa_{i}=0$, with a quasi-period of $1/(2n_{i}d_{i})$, i.e., the half reciprocal of the optical path in the layer. The fringes are widely used for determining either the material’s optical constants or thickness using optical measurements on a bilayer merging a fully transparent while extremely super-wavelength-thick substrate, i.e., one with $d_{i}n_{i}\gg \lambda$, and a layer made of the studied material atop it; see, e.g., Ref. [92].

Resorting to the general multilayer, assume that it contains a layer, *slab*, hereinafter, similar to the substrate in Ref. [92] in terms of thickness but potentially partially transparent. If the slab-derived fringes are too dense, they become useless for characterizing the slab. Moreover, even though the used spectrometer could resolve the fringes, the latter would dominate the optical spectra of the multilayer and thus mask the contribution of other layers, which should be deciphered from the fringes’ envelope. The optical interference in the slab and hence the fringes are destroyed even for one, any pair, or all of the following reasons altogether: (i) the slab thickness $d_{s}$ is slightly randomly nonuniform; (ii) it has the slab bulk refraction index $n_{s}$; (iii) there is a slight incoherence of the used radiation source. For the ideal slab and coherent source, the optical-path phase $\varphi_{s}=Re\left(\delta_{s}\right)=2\pi n_{s}d_{s}/\lambda$, though deterministic, hugely overrides 2π, i.e., makes hugely many cycles. Due to this, although the relative fluctuations in $d_{s}$, $n_{s}$, and λ are small, satisfying the conditions (i)–(iii), any one, any pair, or all, make $\varphi_{s}$ fluctuate at random over many cycles. Due to this, the observable (apparent) reflectivity *R* and transmittance *T*, as self-averaging physical quantities, can be obtained by averaging the ideal ones over $\varphi_{s}$ as follows:

(A10) $$T_{a}=\frac{1}{2\pi }\int_{0}^{2\pi }T\left(\varphi_{s}\right)d\varphi_{s}\;,\;\;R_{a}=\frac{1}{2\pi }\int_{0}^{2\pi }R\left(\varphi_{s}\right)d\varphi_{s}.$$

The above dependences on the optical-path phase in the slab, needed for performing the integration, are obtained with Equation (A9) after solving the iterations given by Equations (A7) and (A8) sequentially.

As an illustration of the technique delineated above, we expose the formulas for one layer (L=1). This example shows how one can obtain the amplitudes $t_{L+1},\;r_{0}$, and so T,R, via Equation (A9), with finite-step recursive algebra, thus avoiding sophisticated Airy infinite-series summation; see, e.g., ([96] Chapter 4), and ([97] Chapter 2). The results read as

(A11) $$T={\left|t_{2}\right|}^{2}={\left|\frac{\tau_{2,1}\tau_{1,0}e^{i\varphi_{1}}}{1+\rho_{0,1}\rho_{1,2}e^{2i\varphi_{1}}}\right|}^{2},\;\;R={\left|s_{0}\right|}^{2}={\left|\frac{\rho_{0,1}+\rho_{1,2}e^{2i\varphi_{1}}}{1+\rho_{0,1}\rho_{1,2}e^{2i\varphi_{1}}}\right|}^{2}.$$

For a slab (1→s) that is partially transparent, i.e., at $n_{s}d_{s}\gg \lambda$ and $n_{s}\gg \kappa_{s}$, processing two terms in Equation (A11) due to the prescription of Equation (A10) yields the apparent transmittance and reflectance as follows:

(A12) $$T_{a}=\frac{{\left(1-R_{s}\right)}^{2}e^{-\alpha_{s}d_{s}}}{1-R_{s}^{2}e^{-2\alpha_{s}d_{s}}},\;R_{a}=R_{s}\left(1+e^{-\alpha_{s}d_{s}}T_{a}\right),$$

where $R_{s}={\left[\left(n_{s}-1\right)/\left(n_{s}+1\right)\right]}^{2}$ is Fresnel’s reflectivity of the slab and $\alpha_{s}=4\pi \kappa_{s}/\lambda$ is the slab’s material absorption coefficient. Equation (A12) is extensively used for thermometric [98], thermal radiation (emissivity) [99,100,101], and Fourier-transform IR spectrometric [91] studies of the semiconductor wafers. Equation (A10) was noted [102] for the first time to be a mathematical procedure, strictly bridging the rigorous Equation (A11) with semi-phenomenological Equation (A12), derived as a rule using semi-intuitive infinite-series summation of the incoherent internal reflection powers; see, e.g., [99] and ([97] Chapter 11).
